# Liposomal bupivacaine versus bupivacaine hydrochloride erector spinae plane blocks in cardiac surgery: protocol for a pragmatic randomized controlled trial

**DOI:** 10.1186/s13063-026-09620-8

**Published:** 2026-04-02

**Authors:** Jin Yoo, Russell J. Pepe, Sorasicha Nithikasem, Kayla N. Laraia, NaYoung K. Yang, Leonard Y. Lee

**Affiliations:** 1 Department of Anesthesiology, Perioperative Care and Pain Medicine, New York University, New York, NY USA; 2 Division of Cardiothoracic Surgery, Department of Surgery, Rutgers Robert Wood Johnson Medical School, New Brunswick, NJ USA; 3 Department of Surgery, Columbia University Irving Medical Center, New York, NY USA; 4 Department of Surgery, Washington University School of Medicine in St. Louis, St. Louis, MO USA; 5 Robert Wood Johnson University Hospital, New Brunswick, NJ USA

**Keywords:** Plane block, Sternotomy, Thoracotomy, Cardiac, Surgery, Postoperative, Pain, Anesthesia, Opioid, Pragmatic trial

## Abstract

**Background:**

Postoperative pain management after cardiac surgery involves a multimodal approach. Recently, the erector spinae plane (ESP) block has been incorporated into multimodal pain control protocols. These measures have been taken to increase patient satisfaction while minimizing opioid usage. Prospective data is lacking to guide decisions regarding optimal regional anesthetic agents. This randomized controlled pragmatic trial protocol seeks to determine the benefit of liposomal bupivacaine relative to plain bupivacaine hydrochloride at reducing postoperative opioid consumption and other clinical outcomes following cardiac surgery.

**Methods:**

The investigators anticipate consenting 150 subjects to obtain 96 evaluable subjects undergoing minithoracotomy (*N* = 24 liposomal bupivacaine, *N* = 24 bupivacaine hydrochloride) or open sternotomy (*N* = 24 liposomal bupivacaine, *N* = 24 bupivacaine hydrochloride). The primary outcome will be postoperative opioid consumption, reported in morphine equivalents. Secondary outcomes will include postoperative nonopioid analgesic consumption, inpatient and outpatient postoperative pain scores, 30-day mortality and major morbidity rates, postoperative quality of life, and hospitalization costs. Double blinding will be conducted with necessary measures taken to mask electronic medical records and drug preparation.

**Discussion:**

The trial is currently enrolling subjects at a single academic medical center in the northeastern United States. The current study aims to investigate the postoperative pain reported by patients undergoing cardiac surgery when receiving ESP blocks with liposomal bupivacaine (experimental) compared to its hydrochloride formulation (control).

**Trial registration:**

ClinicalTrials.gov NCT06077422. Registered on October 2023. https://clinicaltrials.gov/study/NCT06077422?tab=table.

**Supplementary Information:**

The online version contains supplementary material available at 10.1186/s13063-026-09620-8.

## Administrative information

Note: the numbers in curly brackets in this protocol refer to SPIRIT checklist item numbers. The order of the items has been modified to group similar items (see http://www.equator-network.org/reporting-guidelines/spirit-2013-statement-defining-standard-protocol-items-for-clinical-trials/).

**Table:** 

Title {1}	Liposomal bupivacaine versus bupivacaine hydrochloride erector spinae plane blocks in cardiac surgery: protocol for a pragmatic randomized controlled trial
Trial registration {2a and 2b}	Registered on ClinicalTrails.gov: NCT06077422 Other study identification number: Pro2022001580
Protocol version {3}	Protocol version 3, March 2024
Funding {4}	Funding has been provided for this study by Pacira Pharmaceuticals Incorporated.
Author details {5a}	^1^Department of Anesthesiology, Perioperative Care and Pain Medicine, New York University, New York, NY, USA. ^2^Division of Cardiothoracic Surgery, Department of Surgery, Rutgers Robert Wood Johnson Medical School, New Brunswick, NJ, USA. ^3^Department of Surgery, Columbia University College of Physicians and Surgeons, New York, NY, USA. ^4^Department of Surgery, Washington University School of Medicine, St. Louis, MO, USA. ^5^Robert Wood Johnson University Hospital, New Brunswick, NJ, USA.
Name and contact information for the trial sponsor {5b}	Trial Sponsor: Rutgers Robert Wood Johnson Medical School Sponsor’s Reference: Pro2022001580 Contact name: Dr. Leonard Y. Lee, M.D. Address: Rutgers Robert Wood Johnson Medical School 1 Robert Wood Johnson Place East Tower, 8th Floor, Room 843 New Brunswick, NJ 08901 Email: leele@rwjms.rutgers.edu
Role of sponsor {5c}	The trial sponsor is responsible for the protocol design, study conduction, and trial oversight. The study funders will not have any role in study design, collection, management, analysis, and data interpretation. They will have no authority on its manuscript submission or process.

## Introduction

### Background and rationale {6a}

Postoperative pain is a major concern for patients after cardiovascular surgery.

With a growing emphasis on improving perioperative care arising from evidence-based protocols such as Enhanced Recovery After Surgery (ERAS) [1, 2], the matter of reducing postoperative pain is an important topic to target. Mitigation of surgical pain not only increases patient satisfaction [3, 4], but may also decrease postoperative complications [5, 6], augment earlier ambulatory recovery and bowel motility [7, 8], and improve short- and long-term outcomes that are important to patients and programs alike [9–11].

Reduced postoperative pain decreases rates of pneumonia and time on mechanical ventilation [12], time in the intensive care unit (ICU) [13], and hospital length of stay (LOS) [14–16]. Importantly, decreasing postoperative pain through expansion of the nonopioid components of multimodal pain control protocols can also reduce high-dose opioid usage [9, 17], which presents adverse effects including hyperalgesia, respiratory depression, nausea, tolerance, and dependence [18, 19].

Methods of reducing postoperative pain, such as neuraxial anesthesia, deep fascial blocks, and paravertebral blocks, are associated with a relatively increased risk of bleeding, particularly in patients on anticoagulation or antiplatelet therapy [20], or visceral injury due to the deeper trajectory of the block [21, 22]. Studies have shown incidences of hematomas and pneumothorax with paravertebral blocks [23–26]; an alternate solution, therefore, is using fascial plane blocks [27].

Since 2018, our institution has regularly employed preoperative fascial plane blocks for cardiac surgery patients, implementing bilateral plane blocks for the sternotomy approach and unilateral plane blocks for the right minithoracotomy approach. Results from these blocks have been quite favorable anecdotally, but prospective data are lacking. Parasternal nerve blocks target the anterior cutaneous branches of intercostal nerves (T2–T6); erector spinae plane (ESP) blocks cover the dorsal rami and extension into the ventral rami [28]. ESP blocks are considered a safer alternative to parasternal blocks, which are performed adjacent to the internal mammary artery, a potential source of complications, and near the incision site, rendering them less suitable for preoperative injection [29]. Thus, this study evaluates the effectiveness of preoperative ESP blocks for postoperative pain management in cardiac surgery, reflecting a more practical application within an academic institution.

A study in which subjects are randomized to forgo preoperative ESP blocks would raise concerns regarding equipoise (i.e., patients randomized to receive placebo would be subjected to an unreasonable risk of more severe postoperative pain). The authors feel that such concerns create a prohibitive barrier to the design and performance of a placebo-controlled trial comparing liposomal bupivacaine with an inactive control. Therefore, this study is designed to weigh the comparative effectiveness of two Food and Drug Administration (FDA)-approved medications, liposomal bupivacaine (the current practice at our institution) and bupivacaine hydrochloride. Bupivacaine hydrochloride has an onset of action of 2 to 10 min from the time of injection and an effect duration of roughly 7 h [30]. Liposomal bupivacaine is a commercially available, extended-release formulation to up to 72 h from the time of injection [31].

Several studies have compared local injections of bupivacaine hydrochloride with liposomal bupivacaine in the setting of inguinal hernia repair, knee arthroplasty [32, 33], nephrectomy [34], breast augmentation, and hemorrhoidectomy, with results favoring the use of liposomal bupivacaine based on improvements in subjective pain at the initial postoperative pain assessment [35]. Clinical trials are currently underway investigating the effect of liposomal bupivacaine in comparing paravertebral blocks with thoracic epidural blockade in thoracoscopic surgery [36]. In a recent clinical trial published in the *Journal of the American Medical Association* (*JAMA*), patients undergoing cardiothoracic or vascular surgery subjected to truncal incisions did not demonstrate a significant benefit with respect to pain control or adjunctive opioid usage when treated with liposomal bupivacaine over plain bupivacaine via local injection [37]. A randomized controlled trial comparing liposomal bupivacaine with 0.9% saline placebo in parasternal nerve blocks following sternotomy demonstrated only a marginal reduction in pain levels [13]. Still, a gap in the literature exists with respect to the use of liposomal bupivacaine versus plain bupivacaine for ESP blocks in the setting of sternotomy. The aim of this pragmatic randomized controlled trial is to determine the impact of preoperative fascial plane blocks with liposomal bupivacaine compared to bupivacaine hydrochloride in reducing postoperative opioid consumption and subjective pain following cardiac surgery.

### Objectives {7}

The goal of this study is to describe and compare the effectiveness of ultrasound-guided erector spinae plane blocks using Exparel® (bupivacaine liposome injectable suspension) to Marcaine® (bupivacaine hydrochloride) at reducing postoperative opioid consumption, subjective pain scores, quality of life, and clinical outcomes after cardiac surgery.

### Trial design {8}

This study is a pragmatic randomized controlled trial with an active control. It is designed as a phase II/III study with an adaptive design that would allow for the drug delivery protocol to be adjusted as needed in the earliest phase of the trial. Patients enrolled in this initial phase of recruitment would be included in the phase III comparison, provided the protocol for drug delivery is not modified. Enrollment will be stratified into cohorts defined by operative laterality (unilateral ESP block for right minithoracotomy versus bilateral ESP block for median sternotomy) to determine if liposomal bupivacaine is superior to bupivacaine hydrochloride in controlling postoperative pain, defined objectively by postoperative opioid consumption and nonopioid pain medication and subjectively by patient-reported pain scale. Consent will be obtained prior to any study procedures. Subjects will be randomly assigned to receive either liposomal bupivacaine or bupivacaine hydrochloride prior to surgery. Randomization will be done in blocks of 10 (five liposomal bupivacaine and five bupivacaine hydrochloride) within each surgery group (minithoracotomy procedures or open sternotomy). One of 10 sealed envelopes (either liposomal bupivacaine or bupivacaine hydrochloride) will be drawn prior to surgery to determine which drug the subject will receive. Designated study staff (not involved with patient assessments) will randomize and inform the anesthesiologist which medication to administer. The subject and all other study staff will be blinded to treatment group. The anesthesiologist will administer the block per routine care utilizing the designated medication. Surgery will be performed per standard of care. SF-12 questionnaire will be administered and completed prior to surgery and at the routine follow-up clinic visit.

## Methods: participants, interventions, and outcomes

### Study setting {9}

Patients scheduled to undergo elective cardiac surgery at a single academic medical center in the northeastern United States will be screened and consented for participation. In the past year, more than 1500 cardiac surgeries were performed at the institution. The medications involved in this study are readily available and utilized as standard of care.

### Eligibility criteria {10}

#### Brief inclusion criteria

Adults (18 years and older) scheduled for cardiac surgery via minithoracotomy (i.e., valve repair) or open sternotomy (i.e., bypass graft) at a single academic medical center (in- and outpatients). 

#### Brief exclusion criteria

Patients will be excluded if they:

- Are currently on pain medication or a pain regimen for a chronic pain condition
- Convert to sternotomy (for thoracotomies)
- Require, upon intraoperative discovery and surgeon’s decision, the need for an unplanned secondary procedure other than the originally scheduled index operation
- Undergo emergent surgery
- Are non-English speaking (the majority of the expected patient population speaks English. The investigators cannot afford to enroll non-English speaking subjects due to time, personnel, and financial constraints.)
- Require mechanical circulatory support
- Require vasoactive medications
- Are intubated
- Have an active infection
- Are otherwise deemed ineligible for ESP block by the investigators due to safety concerns.

### Who will take informed consent? {26a}

Study physicians or nurses identified on the Institutional Review Board (IRB) will identify potential subjects from the cardiac surgery clinic or inpatient cardiac surgery service.

At the cardiac surgery clinic, the nurse or physician will discuss the study with the subject and give them the written consent to read. All questions will be answered, written consent obtained, and a copy given to the subject. The nurse will follow up if the subject wants to participate.

At the inpatient cardiac surgery service, patients will be approached one or more days prior to surgery in the hospital room. The physician or nurse will discuss the study with the subject, give them written consent to read, and return after the patient has read consent.

Consent and process will be documented in the subject’s medical record as a note by the investigator obtaining consent.

### Additional consent provisions for collection and use of participant data and biological specimens {26b}

Deidentified participant data will be retained for up to 6 years following study completion in accordance with institutional policy and federal Health Insurance Portability and Accountability Act (HIPAA).

regulations. This data may be used for ancillary analyses relevant to the research question, including subgroup analyses and correlational studies. Participants will be informed during their consent that deidentified information may be used for such secondary analyses. Biological specimens will not be collected.

## Interventions

### Explanation for the choice of comparators {6b}

The active comparator medication (bupivacaine hydrochloride) was selected to compare the typical hydrochloride form of bupivacaine to our interventional liposomal formulation in local blocks. There is continued debate about the usage of hydrochloride versus liposomal bupivacaine, with aspects to consider such as pain control to differences in costs.

### Intervention description {11a}

The experimental medication (bupivacaine liposome injectable suspension) will be administered per standard of care via ultrasound-guided ESP blocks: bilaterally for sternotomy and unilaterally for minithoracotomy (liposomal bupivacaine 133 mg/10 mL, liposomal bupivacaine 266 mg/20 mL ± expansion with saline). The drug will be administered prior to cardiac surgery. The dosage will be determined by patient body weight.

The investigators anticipate consenting 120 participants to obtain 96 evaluable subjects: 48 minithoracotomies (*N* = 24 liposomal bupivacaine, *N* = 24 bupivacaine hydrochloride) and 48 open sternotomies (*N* = 24 liposomal bupivacaine, *N* = 24 bupivacaine hydrochloride).

### Criteria for discontinuing or modifying allocated interventions {11b}

N/A: No adverse effects are anticipated, given that the use of ESP blocks has been routine at this facility for several years.

### Strategies to improve adherence to interventions {11c}

N/A: Patient adherence is not necessary for this study.

### Relevant concomitant care permitted or prohibited during the trial {11d}

N/A: There is no concomitant care that is prohibited during the trial.

### Provisions for post-trial care {30}

N/A: No provisions for harm are anticipated given that the use of ESP blocks has been routine at this facility for several years.

### Outcomes {12}

Data points collected are included in the attached study protocol in the supplemental materials (see Additional file 1). The primary outcome is postoperative opioid consumption within the first five postoperative days, reported as average mg/day in morphine equivalents. The time frame for this outcome is within postoperative days 0 through 5.

Secondary outcomes measured within postoperative days 0 through 5 include postoperative nonopioid analgesic consumption and average inpatient postoperative pain score (1, least severe, to 10, most severe).

Secondary outcomes measured within postoperative days 0 through 30 include outpatient postoperative pain score, 30-day mortality rate, and 30-day major morbidity rate. Postoperative pain scores will be reported as mean pain score, compared between groups using independent-sample *t*-tests or Mann–Whitney *U* test (if non-normal distribution). Thirty-day mortality rate and 30-day major morbidity rate will be metrics compared between groups using Fisher’s exact test or chi-square test.

Postoperative quality of life will be measured using the 12-item Short Form Survey (SF-12) from preoperative baseline to follow-up within 30 days after surgery, analyzed as mean change with independent-samples *t*-test or Mann–Whitney *U* test. Initial scoring will be conducted within the range of 30 days prior to surgery. Scores on the SF-12 range from 0 to 100, with higher scores indicating better physical and mental health.

Hospitalization cost will be derived before-insurance at the study duration, limited to 1 year. It will be reported as mean cost per patient, compared between groups with independent-samples *t*-test or Mann–Whitney *U* test.

### Participant timeline {13}

**Fig. 1:**
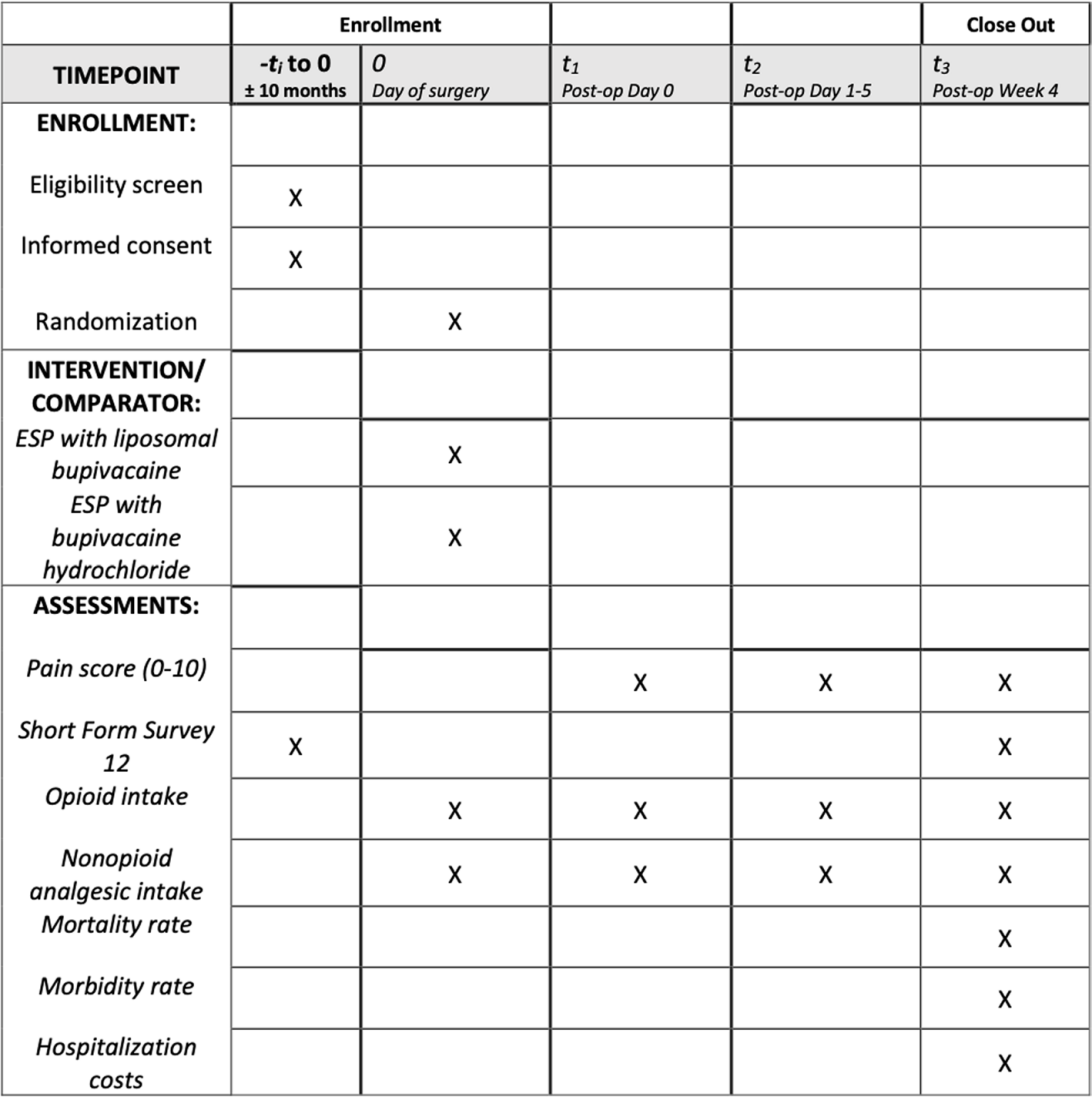
SPIRIT participant timeline

Patients will follow the timeline as seen in Fig. 1, with trial enrollment at the preoperative clinic visit days to weeks prior to surgery, followed by monitoring throughout their hospital stay and at postoperative week 4.

### Sample size {14}

The investigators anticipate consenting 150 subjects for screening to obtain 96 evaluable subjects, accounting for screening failures, i.e., exclusion criteria; 48 minithoracotomies (*N* = 24 liposomal bupivacaine, *N* = 24 bupivacaine hydrochloride) and 48 open sternotomies (*N* = 24 liposomal bupivacaine, *N* = 24 bupivacaine hydrochloride).

**Fig. 2:**
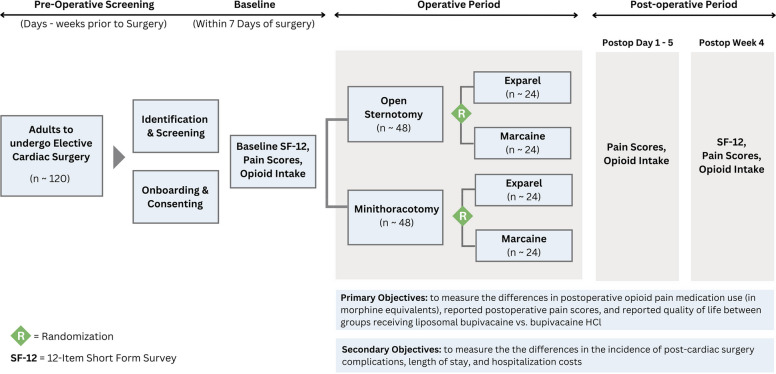
Study sample selection and randomization timeline

Patients will be randomized using block randomization within each surgery group (minithoracotomy procedures or open sternotomy) for a total of 24 patients per group or drug combination (48 minithoracotomies (24 liposomal bupivacaine, 24 bupivacaine hydrochloride) and 48 open sternotomies), as seen in Fig. 2.

We based our study on a power analysis conducted with approximated means from Song et al. [38]. The sample size of their experimental group was 8, but the study design was the first to include liposomal bupivacaine, so we used their reported data to inform our power calculation. Their reported means and standard deviations for postoperative opioid consumption at 12 h were 0.203 ± 0.097 mg/kg for the liposomal bupivacaine intervention group versus 0.334 ± 0.094 mg/kg for the control group, corresponding to an effect size of Cohen’s *d* = 1.37. Using a 2-sided alpha of 0.05 and a power of 0.80, we calculated that 12 patients per group would be required. To account for both surgical approaches and allow for subgroup analyses, we doubled this to 24 patients per treatment arm within each surgical subgroup, yielding 96 total evaluable patients. This calculation was performed using Statistical Analysis System (SAS) software.

**Fig. 3:**
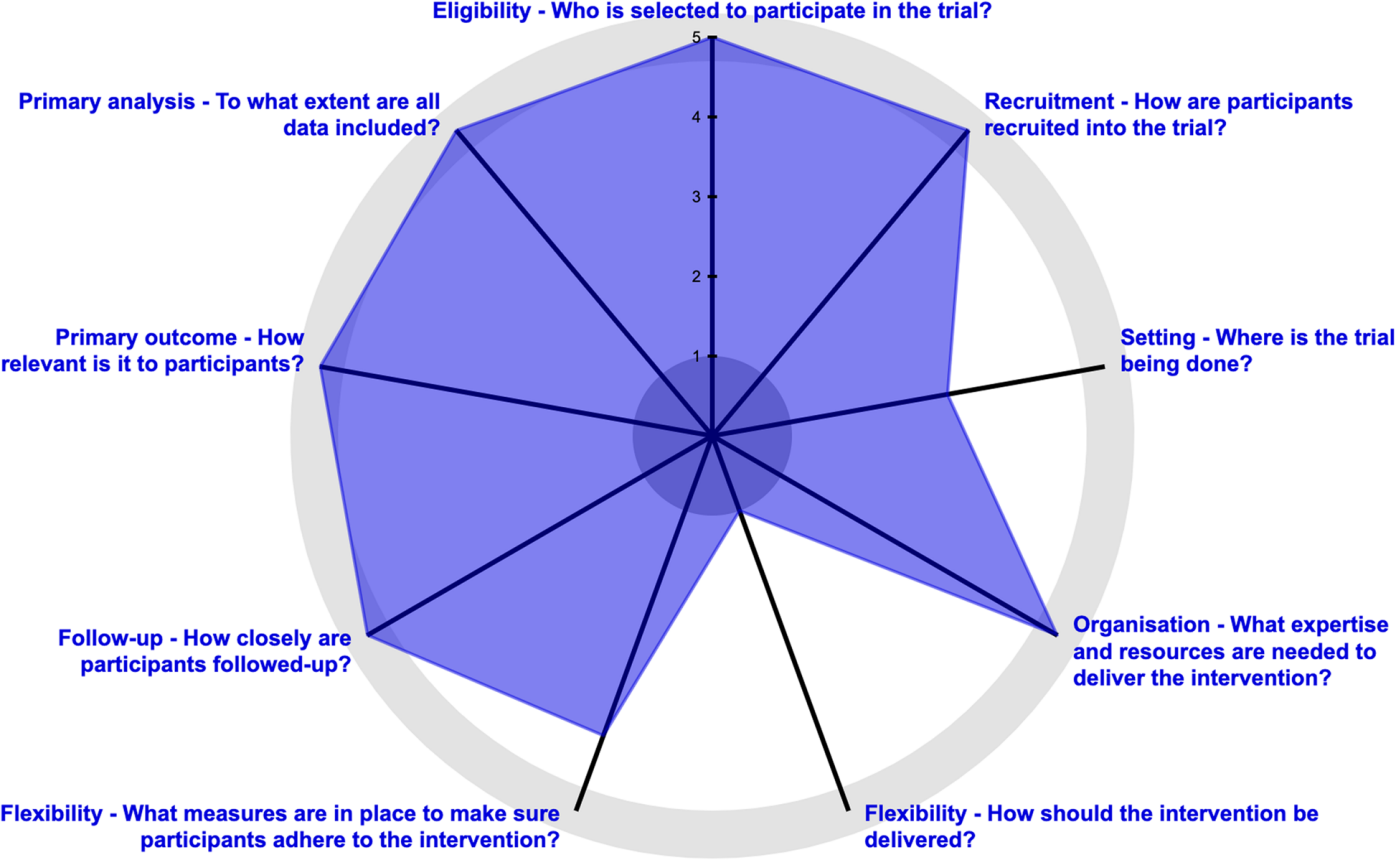
PRECIS-2 model to assess the efficacy of preoperative ESP blocks on cardiac surgery postoperative outcomes

We created a PRagmatic-Explanatory Continuum Indicator Summary (PRECIS-2) model (Fig. 3) to analyze the domains of eligibility criteria, recruitment, setting, organization, flexibility delivery, flexibility adherence, follow-up, primary outcome, and primary analysis. Each domain is examined from 1 to 5, based on a scale representing how explanatory (1) to pragmatic (5) the trial is, with rim proximity indicating (5) the most pragmatic approaches.

Our model, shown in Fig. 3, scored a 5 in eligibility, recruitment, organization, follow-up, primary outcome, and primary analysis. The flexibility and setting scores ranged from 1 to 4. This indicates an overall pragmatic trial design.

### Recruitment {15}

Potential subjects will be recruited from the cardiac surgery clinic and/or service (i.e., inpatients) day(s) or weeks prior to surgery. Our institution averages about 1700 cases annually. Out of these cases, the majority are elective surgeries that require preoperative clinic visitations, at which patients will be evaluated for possible recruitment. There is low concern for achieving adequate participant enrollment to reach the target sample size, which is only around 5% of the annual case load.

## Assignment of interventions: allocation

### Sequence generation {16a}

Randomization will occur with a computerized random number generator set to create equal distribution within (A) liposomal bupivacaine and (B) bupivacaine hydrochloride, stratified by surgical approach (minithoracotomy versus open sternotomy). Block randomization will be used to ensure balance within each stratum.

Each randomization assignment will be printed on individual sheets of paper. Each sheet will be folded individually into sealed envelopes that will be labeled with the patient’s study identification number upon enrollment.

### Concealment mechanism {16b}

The allocation sequence will be concealed using sequentially numbered, sealed envelopes. Each envelope will be prepared in advance by the trial manager (who is not involved in patient recruitment or clinical care). They will contain the treatment allocation for each participant and remain locked in a cabinet accessible only to the trial manager and the designated cardiac anesthesiologist administering the block. Upon enrollment, each participant will be assigned the next sequential envelope number based on their surgical strata. The envelope will be sealed until the day of surgery, at which time the cardiac anesthesiologist will be unblinded to administer the medication. Notably, the anesthesiologist administering the ESP block will be resourced solely for that purpose and will not remain with the patient during the operation nor during the postoperative transfer of care to the cardiovascular ICU, preserving the effect of blinding with respect to the study personnel recording subjective pain scores and administering intra- and postoperative pain medications.

### Implementation {16c}

The trial manager will generate the allocation sequence.

The participating attending surgeons will introduce the study and, if applicable, receive consent to patients who meet inclusion criteria. The certified clinical research associate will oversee the enrollment of participants and their intervention assignment.

## Assignment of interventions: blinding

### Who will be blinded {17a}

The design of the study implements purposeful protocols to maintain blinding throughout the study. In designing this protocol, several obstacles needed to be addressed.

First, the matter of drug administration posed a problem, as bupivacaine hydrochloride is clear and liposomal bupivacaine is opaque. Thus, a cardiac anesthesiologist not assigned to the operation will prepare and administer the block. To maximize blinding, the syringe containing the bupivacaine will be opaque, so those around the anesthesiologist administering the ESP block will be blinded in the study. The cardiac anesthesiologist assigned to perform the sedation and maintenance during surgery will remain blinded to the study.

Second, the nurses in charge of the postoperative care of the patient have access to their medical records, which document which drug the patient had received. With the knowledge of which drug they received, the nurses and other caregivers would be subject to observer bias. To work around this, the participants of the trial will have electronic medical records that state they are taking part in a clinical trial in which either medication could have been administered, without specifying which was given.

**Fig. 4:**
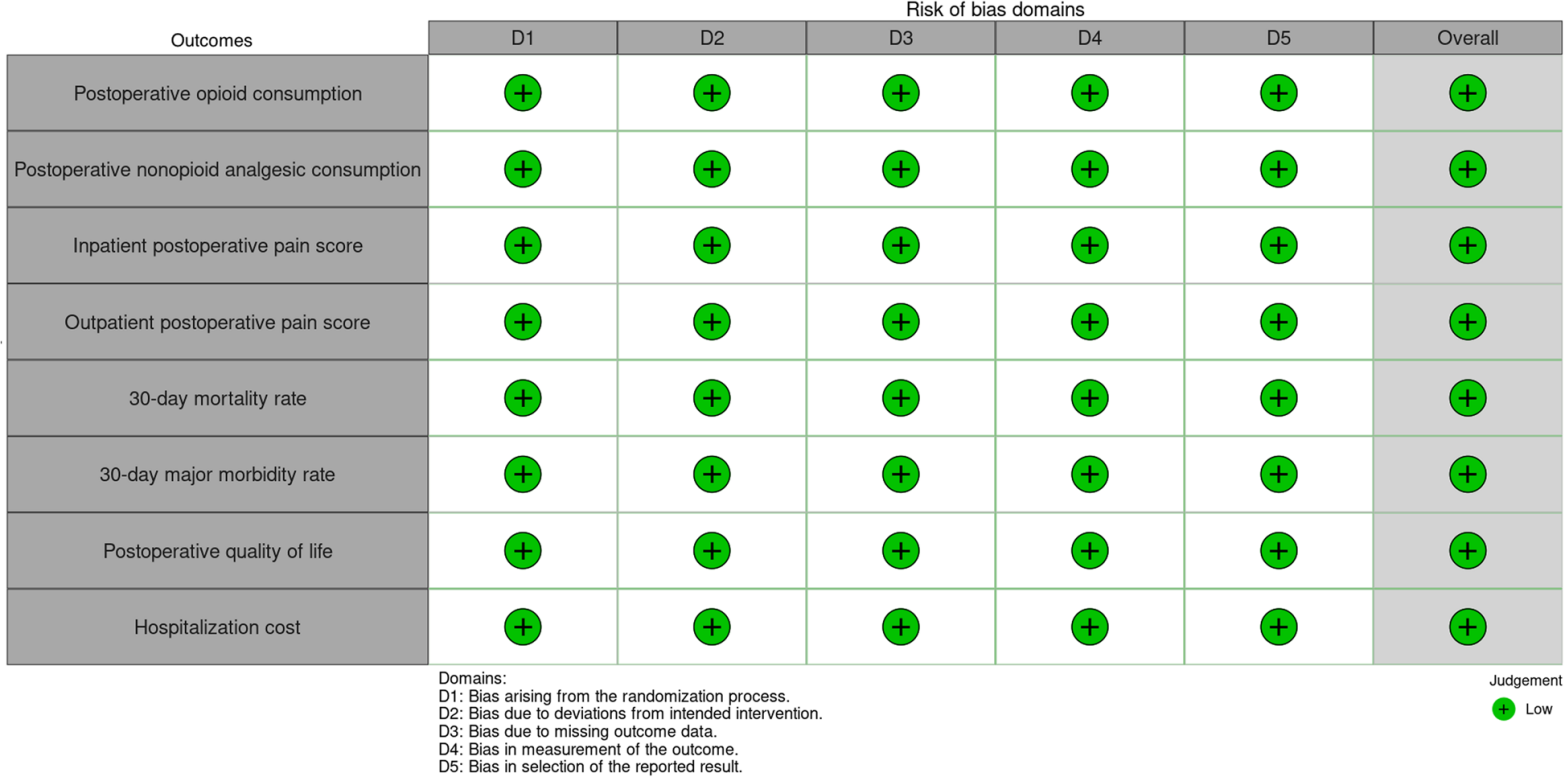
Cochrane risk of bias assessment to evaluate preoperative ESP blocks on cardiac surgery postoperative outcomes [39]

The analysis of these measures will be performed according to an intention-to-treat protocol. Our outcomes were reviewed with the Cochrane risk-of-bias tool. Figure 4 shows our Risk of Bias, which found low risk for all the outcomes (postoperative opioid consumption, postoperative nonopioid analgesic consumption, inpatient postoperative pain score, outpatient postoperative pain score, 30-day mortality rate, 30-day major morbidity rate, postoperative quality of life, hospitalization cost).

### Procedure for unblinding if needed {17b}

Unblinding in the case of any safety or other concerns is possible by referring to the trial manager’s records and to the anesthesiologist administering the drug preoperatively.

## Data collection and management

### Plans for assessment and collection of outcomes {18a}

Outcomes including hospital LOS, costs, pain scores, and postoperative opioid and nonopioid analgesic medication consumption will be obtained via chart review. Opioid use will be reported as oral morphine equivalents, as variables will vary in mode of administration and type of opioid. The measurements will be taken as a total volume used during the hospital stay and during the weeks following discharge until the 4-week follow-up appointment. Pain scores from 1 to 10 (10 most severe), obtained by the ICU and floor nurses, will be averaged daily through postoperative day five and at the first follow-up appointment (4 weeks after discharge). Other outcomes will be assessed at the follow-up visit within 30 days after the surgery.

### Plans to promote participant retention and complete follow-up {18b}

All cardiac surgery patients at our institution routinely undergo a 4-week postoperative follow-up. Based on institutional experience, loss to follow-up at this time point is expected to be minimal. Because study follow-up is akin to regular postoperative care, we anticipate high participant retention.

### Data management {19}

Baseline information will be collected during hospitalization and in the SF-12 forms given at clinic. Pre-, peri-, and postoperative information will be in an online platform, and data are input and stored into protected and confidential online spreadsheets after manual data collection from electronic medical records. The trial manager and certified clinical research associate will overlook the data quality and validation of the study database.

### Confidentiality {27}

Data will be entered into the REDCap study database. REDCap is a database program purchased by the institution for use in research. The study database is maintained on the secure institutional server, password protected and only accessible to the designated research staff. Upon completion of the study, all identifiers in the database will be removed and stored by study-specific identification number only. Once this is done, there will be no link between the subject and health information. Deidentified data will be stored for 6 years in accordance with institutional requirements.

### Plans for collection, laboratory evaluation and storage of biological specimens for genetic or molecular analysis in this trial/future use {33}

N/A: No biological specimens will be collected in this study.

## Statistical methods

### Statistical methods for primary and secondary outcomes {20a}

We will conduct a *t*-test (assuming normal distribution) on our randomized sample selection for our continuous variables, and a Fisher’s exact or chi-square (the former if *N* < 10) for categorical variables. We will initially compare our sample descriptive data to scale the range of diversity within and between our two groups.

After describing our sample, we will compare our continuous dependent variables (postoperative opioid and nonopioid analgesic consumption, inpatient and outpatient pain scores, length of stay in the hospital, etc.) with *t*-tests. We will repeat these calculations within strata by operation type.

### Interim analyses {21b}

We do not anticipate the need for a formal interim analysis for safety concerns, as this trial continues along the lines of routine clinical care. Any adverse events assumed to be directly linked to the experiment will be treated appropriately, recorded, and placed into consideration of study modification. Research assistants, nurse research manager, and attending physicians involved in surgery or block administration will be notified of any adverse events.

### Methods for additional analyses (e.g., subgroup analyses) {20b}

The subgroup analysis of this study will be based on method of incision/approach: minithoracotomy versus full sternotomy.

### Methods in analysis to handle protocol non-adherence and any statistical methods to handle missing data {20c}

All analyses will follow the intention-to-treat design, where participants will be analyzed according to their assigned treatment assignment, regardless of the actual intervention received or potential protocol deviation, to minimize attrition bias. Due to the nature of a single intervention administered at a fixed time point (preoperatively), we do not anticipate issues with treatment adherence. Any protocol deviations will be documented and reported in further publications. We do not anticipate substantial missing data due to the routine documentation of postoperative opioid consumption in the electronic medical record for all cardiac surgery patients. However, in the event of any missing data from incomplete documentation or participant withdrawal, we will examine the patterns of missingness (missing completely at random, at random, or not at random) to implement appropriate multiple imputation methods based on complete participant data. In this event, we will clearly report how much data was missing.

### Plans to give access to the full protocol, participant level-data and statistical code {31c}

Further access for trial protocol, deidentified data, and statistical codes can be made with direct contact to the research group. The principal investigator will provide access in accordance with the institutional policies and Good Clinical Practice guidelines.

## Oversight and monitoring

### Composition of the coordinating center and trial steering committee {5d}

The coordinating center includes all those involved in the trial. It is made up of the principal investigator, attending cardiothoracic surgeons, attending cardiac anesthesiologists, a certified nurse research coordinator, a statistician, and a team of trial investigators made up of medical students and general surgery residents. The attending cardiothoracic surgeons will perform cardiac surgeries, and the cardiac anesthesiologists will administer the ESP blocks. The nurse research coordinator will handle the oversight of data collection. The statistician will perform data analysis and interpretation. The trial investigators will guide the study’s preparation, presentation, publication, and fiscality. The principal investigator will oversee all operations and necessary communication between parties. The group will meet weekly to exchange updates on the trial, with any immediate communication also possible daily.

### Composition of the data monitoring committee, its role and reporting structure {21a}

There is no need for a data monitoring committee on this trial, as there is very low suspicion for the potential to harm patients with either control or experimental interventions.

### Adverse event reporting and harms {22}

Patients at this hospital normally receive liposomal bupivacaine if they undergo an ESP block. Both study medications are FDA-approved local anesthetics routinely used in clinical practice. Therefore, the primary risk specific to this study is if one medication is less effective at controlling pain than the other, which would be managed per standard postoperative analgesic protocols. While generally safe when performed under ultrasound guidance, ESP blocks can lead to pneumothorax, infection, bleeding, and local anesthetic systemic toxicity. Both local anesthetics, while FDA-approved, have the risk of common side effects including nausea, vomiting, back pain, and dizziness [30, 31]. If any life-threatening conditions occur from block injection or medication effects, the adverse event will be documented as a serious adverse event (SAE). These events will be systematically collected and investigated by the data safety monitoring board to determine if the circumstance was causally related to the study intervention. Any SAE will be reported if there is a causal relationship to the intervention and control ESP block. SAEs will be reported in the Medical Dictionary for Regulatory Activities (MedDRA) language.

### Frequency and plans for auditing trial conduct {23}

N/A: Auditing trial conduct is not expected, as all operations undergo extensive supervision as part of routine clinical care.

### Plans for communicating important protocol amendments to relevant parties (e.g., trial participants, ethical committees) {25}

If such amendments, such as inclusion criteria, secondary outcomes, or analyses, must be made, the investigators will provide full transparency to trial participants, registries, potential oral or written bodies of work, and all other key stakeholders.

### Dissemination plans {31a}

The investigators plan to communicate trial protocol and results to healthcare professionals and the public via conferences and peer-reviewed journals.

## Discussion

### Goals

Currently, there are no randomized studies investigating the outcomes of pain medication usage after unilateral or bilateral ESP blocks with liposomal bupivacaine prior to cardiac surgery [40]. Athar et al. performed a randomized controlled trial on the effects of ultrasound-guided bilateral ESP blocks with 0.25% levobupivacaine, the pure S(-)-enantiomer of bupivacaine hydrochloride, compared to saline prior to induction [41]. Similarly, Krishna et al. performed a single-blinded randomized controlled trial on the usage of bilateral ESP blocks with 0.375% ropivacaine [42]. The only trial that uses liposomal bupivacaine is a case-control study by Song et al., a case-control study that compared patients with ESP blocks to those who did not receive a block [38]. While there are recent randomized controlled studies using liposomal bupivacaine in local incision anesthesia and paravertebral blocks, none have been published on ESP blocks, which target multilevel rami while avoiding arterial structures and can be performed preoperatively without impacting the surgical site. Our study aims to establish evidence-based pain control on the usage of ESP blocks with varying forms of bupivacaine.

To ensure validation of a widely popularized mode of anesthetic, we are enrolling a double-blinded, randomized controlled trial for patients undergoing cardiac surgery. Stratified by surgical approach (median sternotomy or unilateral right minithoracotomy), patients will be given an ESP block, consisting of bupivacaine in either the liposomal or hydrochloride formation. We will measure the postoperative pain scores across both groups to determine whether there is a difference in pain assessment from the patient’s perspective with the goal to improve overall patient satisfaction, comfort, and recovery. We will monitor all pain-relief medication use, measured by opioid and nonopioid equivalents to compare drug usage. Investigating and calibrating pain management protocols after cardiac surgery is essential for pain anesthesiology and the surgical team to provide effective patient care and recovery. We hope that the results of our study can help guide changes that decrease postoperative pain, increase patient comfort and quality of life, and minimize adverse outcomes and fiscal characteristics.

### Challenges

Some foreseeable risks of this study include the common side effects of the medications. Some common side effects of bupivacaine hydrochloride include nausea, vomiting, chills, shivering, headache, back pain, dizziness, problems with sexual function, restlessness, anxiety, dizziness, tinnitus, blurred vision, tremor [30]. Side effects of liposomal bupivacaine include dizziness, drowsiness, nausea, constipation, vomiting, itching, headache, back pain, or swelling in hands/feet [31]. Both medications are FDA approved for the indication.

There were considerations made to ensure proper blinding and randomization. While measures were taken, there is a chance that accidental unblinding could occur during the handoff process. Any event of unblinding or non-randomization will be documented and handled consistently with any other events. Lastly, due to the follow-up process of this study, there is a possibility that patients will be lost to follow-up.

## Conclusions

This protocol outlines the design of a pragmatic, double-blinded randomized controlled trial comparing preoperative ESP blocks with liposomal bupivacaine versus bupivacaine hydrochloride in patients undergoing cardiac surgery via minithoracotomies and standard sternotomies. To our knowledge, this trial represents the first randomized controlled trial to directly compare these two formulations in the setting for ESP blocks for cardiac surgery. This trial seeks to generate evidence that can guide clinical decision-making and inform cost-effective pain management strategies in the care of this complex patient population.

## Trial status

This study is a phase II and III interventional study, assessing the effects of bupivacaine hydrochloride versus liposomal bupivacaine. The trial was first submitted to be registered on ClinicalTrials.gov in September 2023 (NCT06077422). The current manuscript represents the first formal, peer-reviewed protocol submission to a scientific journal, submitted in September 2025. Participant recruitment began on January 11th, 2024, anticipated to conclude on January 11th, 2026. The study completion date is estimated for April 5th, 2026. The delay in manuscript submission was due to extended IRB modification cycles, staffing constraints, and negotiations with the funding body.

## Supplementary Information

Additional file 1. Randomized Control Trial to Assess the Efficacy of Preoperative Erector Spinae Blocks on Cardiac Surgery Postoperative Outcomes Protocol # 2022001580. Institutional IRB Protocol provides details on exact data points collected; see pages 5–7.

## Data Availability

The final trial dataset will be available to individual trial investigators who have no patient-facing interaction with the participants but require a need to manage the data including the trial manager, the certified clinical research associate, and the statistician. Other investigators, who have direct contact with participants, will be limited from accessing the final trial dataset until analysis has been conducted.

## Abbreviations

CABG: Coronary artery bypass grafting
ESP: Erector spinae plane
FDA: Food and Drug Administration
HIPAA: Health Insurance Portability and Accountability Act
IRB: Institutional Review Board
ICU: Intensive care unit
ICMJE: International Committee of Medical Journal Editors
*JAMA*: *Journal of the American Medical Association*
LOS: Length of stay
MedDRA: Medical Dictionary for Regulatory Activities
N/A: Not applicable
SAE: Serious adverse event
SAS: Statistical Analysis System
SF-12: Short Form Survey
