# Supplementary material for: Liposomal bupivacaine versus bupivacaine hydrochloride erector spinae plane blocks in cardiac surgery: protocol for a pragmatic randomized controlled trial
Source: Trials. 2026 Apr 2;27:270. doi: 10.1186/s13063-026-09620-8 (PMC13045092; doi:10.1186/s13063-026-09620-8)
Supplement: Supplementary file 1 — Additional file 1. Randomized Control Trial to Assess the Efficacy of Preoperative Erector Spinae Blocks on Cardiac Surgery Postoperative Outcomes Protocol # 2022001580. Institutional IRB Protocol provides details on exact data points collected; see pages 5–7. [file 13063_2026_9620_MOESM1_ESM.docx]

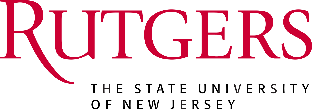


**INTERVENTIONAL**

**RESEARCH PROTOCOL TEMPLATE**

**(HRP-503a)**

**Randomized Control Trial to Assess the Efficacy of Preoperative Erector Spinae Blocks on Cardiac Surgery Postoperative Outcomes**

**Protocol # 2022001580**

**Primary Investigator:**

**Leonard Y. Lee, MD**

Chair, Department of Surgery

Telephone: 732-235-8725

Email Address: leele@rwjms.rutgers.edu

| **Table of Contents**  Skip To Section: Hold **CTRL** + **Click (Below)** To Follow Link in **Blue** | | | |
| --- | --- | --- | --- |
|  | **1.0** | [Research Design](#one0) |  |
|  | 1.1 | [Purpose/Specific Aims](#one1) |  |
|  | 1.2 | [Research Significance](#one2) |  |
|  | 1.3 | [Research Design and Methods](#one3) |  |
|  | 1.4 | [Preliminary Data](#one4) |  |
|  | 1.5 | [Sample Size Justification](#one5) |  |
|  | 1.6 | [Study Variables](#one6) |  |
|  | 1.7 | [Drugs/Devices/Biologics](#one7) |  |
|  | 1.8 | [Specimen Collection](#one8) |  |
|  | 1.9 | [Data Collection](#one9) |  |
|  | 1.10 | [Timetable/Schedule of Events](#one10) |  |
|  | **2.0** | [Project Management](#two0) |  |
|  | 2.1 | [Research Staff and Qualifications](#two1) |  |
|  | 2.2 | [Research Staff Training](#two2) |  |
|  | 2.3 | [Other Resources](#two3) |  |
|  | 2.4 | [Research Sites](#two4) |  |
|  | **3.0** | [Multi-Center Research](#three0) |  |
|  | **4.0** | [Subject Considerations](#four0) |  |
|  | 4.1 | [Subject Selection and Enrollment Considerations](#four1) |  |
|  | 4.2 | [Obtaining Identifiable Information About Non-Subjects](#four2) |  |
|  | 4.3 | [Number of Subjects](#four3) |  |
|  | 4.4 | [Consent Procedures](#four4) |  |
|  | 4.5 | [Special Consent Populations](#four5) |  |
|  | 4.6 | [Economic Burden and/or Compensation For Subjects](#four6) |  |
|  | 4.7 | [Risks of Harm/Potential for Benefits to Subjects](#four7) |  |
|  | **5.0** | [Special Considerations](#five0) |  |
|  | 5.1 | [Health Insurance Portability and Accountability Act (HIPAA)](#five1) |  |
|  | 5.2 | [Family Educational Rights and Privacy Act (FERPA)](#five2) |  |
|  | 5.3 | [Code of Federal Regulations Title 45 Part 46 (Vulnerable Populations)](#five3) |  |
|  | 5.4 | [General Data Protection Regulation (GDPR)](#five4) |  |
|  | 5.5 | [NJ Access to Medical Research Act (Surrogate Consent)](#five5) |  |
|  | **6.0** | [Data Management Plan](#six0) |  |
|  | 6.1 | [Data Analysis](#six1) |  |
|  | 6.2 | [Data Security](#six2) |  |
|  | 6.3 | [Data Safety And Monitoring](#six3) |  |
|  | 6.4 | [Reporting Results](#six4) |  |
|  | 6.5 | [Secondary Use of Data](#six5) |  |
|  | **7.0** | [Research Repositories – Specimens and/or Data](#seven0) |  |
|  | **8.0** | [Approvals/Authorizations](#eight0) |  |
|  | **9.0** | [Bibliography](#nine0) |  |

| **1.0 Research Design** |
| --- |

**1.1 Purpose/Specific Aims**

The goal of this pilot study is to describe and compare Ultrasound-Guided Erector Spinae Plane (ESP) Blocks using Exparel® (bupivacaine liposome injectable suspension) to Marcaine® (bupivacaine hydrochloride) for pain management and outcomes after cardiac surgeries.

**A. Objectives**

To assess and compare patients treated with Exparel vs. Marcaine on;

- postoperative opioid usage,
- postoperative subjective pain scale
- postoperative nonopioid analgesic usage
- composite adverse outcomes, encompassing all-cause mortality and major morbidity (stroke, MI, respiratory failure, kidney failure)
- postoperative quality of life

**B. Hypotheses / Research Question(s)**

Compared to Marcaine patients treated with Exparel will have:

- less usage of opioid pain medications
- noninferior rates of major adverse outcomes, including morality and major morbidity
- lower postoperative pain scores
- less usage of nonopioid analgesics
- improved quality of life

**1.2 Research Significance**

Postoperative pain is a major concern for patients after cardiovascular surgery (1). With an increasing emphasis on improving perioperative care arising from evidence-based protocols such as Enhanced Recovery After Surgery (ERAS), reducing postoperative pain not only increases patient satisfaction, but also decreases postoperative complications and improves outcomes. Reduced postoperative pain also decreases rates of pneumonia and time on mechanical ventilation, in the ICU, or in the hospital (2). Importantly, decreasing postoperative pain can also reduce high dose opioid usage, the established method of managing postoperative pain, thereby decreasing rates of nausea/vomiting, intubation time, and mortality (3). Methods of reducing postoperative pain, such as neuraxial anesthesia and deep plexus blocks, are associated with an increased risk of epidural hematoma; an alternate solution, therefore, is using fascial plane blocks (4).

Since 2018, our institution is one of very few that uses pre-operative fascial plane blocks for cardiac surgery patients, implementing bilateral plane blocks for the sternotomy approach and unilateral plane blocks for the right mini-thoracotomy approach. Results from these blocks have been quite favorable but not yet validated. A study in which subjects are randomized to not receive an ESP block preoperatively would raise concerns regarding equipoise.Therefore, this study is designed to better understand the overall effects of these blocks by comparing FDA approved medications, Exparel (current practice at RWJUH) and Marcaine. Marcaine (0.25% bupivacaine HCl) has an onset of action on the order of seconds and generally within one minute from the time of injection, and an effect duration of roughly seven hours.(5) Exparel is a commercially available extended-release formulation of liposomal bupivacaine, which extends the effect duration to an upward of 72 hours from the time of injection with similarly immediate onset of action.(6) Several studies have compared local injections of Marcaine (plain bupivacaine) with Exparel (liposomal bupivacaine) in the setting of inguinal hernia repair, knee arthroplasty, breast augmentation, and hemorrhoidectomy, with results favoring the use of liposomal bupivacaine based on improvements in subjective pain at the initial postoperative pain assessment.(6) Clinical trials are currently underway, investigating the effect of liposomal bupivacaine for local anesthesia and compared with epidural anesthesia in thoracoscopic surgery.(7,8) In a recent clinical trial published in *JAMA*, patients undergoing cardiothoracic or vascular surgery subjected to truncal incisions did not demonstrate a significant benefit with respect to pain control or adjunctive opioid usage when treated with liposomal bupivacaine over plain bupivacaine via local injection.(9) Still, a gap in the literature exists with respect to the use of liposomal bupivacaine versus plain bupivacaine for erector spinae plane block in the setting of sternotomy. The aim of this prospective study is to determine the impact of preoperative fascial plane blocks with Exparel compared to Marcaine on outcomes following cardiac procedures.

**1.3 Research Design and Methods**

1. **Research Procedures**

This study is a prospective, randomized, controlled trial.

Consent will be obtained prior to any study procedures.

The subject will be randomly assigned to receive either Exparel or Marcaine during surgery.

Randomization will be done in blocks of 10 (5 Exparel and 5 Marcaine) within each surgery group (Mini thoracotomy procedures (Minis) or Open sternotomy (Open).

One of 10 sealed envelopes (either Exparel or Marcaine) will be drawn prior to surgery to determine which drug the subject will receive.

Designated study staff (not involved with patient assessments) will randomize and inform the anesthesiologist which medication to administer via the block. The subject and all other study staff will be blinded to treatment group.

The anesthesiologist will administer the block per routine care utilizing the designated medication.

Surgery will be performed per standard of care.

SF-12 questionnaire will be administered/completed prior to surgery and at the routine follow-up clinic visit.

1. **Data Points**

| Study ID |  |
| --- | --- |
| Exparel group? |  |
| Age |  |
| Gender |  |
| Ethnicity |  |
| BMI |  |
| Race |  |
| Admit Date |  |
| Discharge Date |  |
| LOS |  |
| LOS ICU |  |
| Time of intubation |  |
| Time of extubation |  |
| Intubation time |  |
| Primary operating surgeon |  |
| Case booking status | Elective or Urgent(diagnosis & surgery occurring in same hospitalization) |
| Hospitalization costs |  |
| **Co-morbidities:** |  |
| inflammatory disorders (list) |  |
| pre-existing arrhythmias | atrial or ventricular |
| Hypertension, dyslipidemia, DM | MI, Pre-op pacemaker/defibrillator, CVA |
| **Smoking History** | STS and NYHA score |
| **Outcomes (through 30 days)** |  |
| Pneumonia |  |
| Respiratory failure with ventilatory support >48h |  |
| Pulmonary embolism |  |
| DVT |  |
| Organ Failure |  |
| Post-op MI | clinical dx, cardiogenic shock |
| Acute renal failure requiring renal replacement therapy | Dialysis CVVH |
| Cerebral Vascular Accident (CVA) |  |
| Infection | wound or line & UTI |
| Readmission |  |
| Post op Arrhythmia | Atrial, ventricular |
| New Post-op A-fib | Post op pacemaker/defibrillator |
| Exacerbation of Pre-existing A-Fib |  |
| **Discharge Status:** |  |
| Home, Rehab or Expired |  |
| **Pain scores thru postop d 5** | Daily Average |
| **One time pain score at f/u visit** | One score |
|  |  |
| **In-hospital opioid usage (dose mg/day)** | thru postop d 5 |
| PCA Dilaudid (Hydromorphone) | convert all to morphine |
| IV Dilaudid |  |
| Oral Dilaudid |  |
| PCA Fentanyl |  |
| IV Fentanyl |  |
| Oral Fentanyl |  |
| Opioid start and stop dates |  |
| morphine, oxycodone, percocet/any combined meds |  |
| codeine, tylenol #3 |  |
|  |  |
| **In-hospital non-opioid usage** | thru postop d 5 |
| Lyrica (Pregabalin) |  |
| Gabapentin |  |
| Toradol (Ketorolac) |  |
| Tramadol |  |
| Motrin (Ibuprofin) |  |
| SSRI/SNRI |  |
| Acetaminophen |  |
| non- Opioid start and stop dates |  |
|  |  |
| **SF-12 scores (baseline and f/u)** |  |

1. **Study Duration**

Subject participation will be through postoperative clinic visit (approximately 4 weeks after surgery).

It will take approximately 2 years to complete this study.

1. **Endpoints**

Upon completion of enrollment, data will be analyzed to determine feasibility of proceeding with a larger trial. Based on the results of our preliminary trial in a small cohort of patients, the investigators will expand enrollment in the study to the predetermined sample size expected to be sufficient to achieve statistical significance. In order to calculate this number of study subjects needed to detect a statistically significant difference between the Exparel and plain Marcaine groups, a lowest *clinically* significant difference in postoperative opioid utilization was referenced from existing literature. (See power calculation.)

**1.4 Preliminary Data** N/A

**1.5 Sample Size Justification**

Primary outcome is postoperative opioid use. Secondary outcome is pain scores.

Power calculations are based on [this study](https://nam02.safelinks.protection.outlook.com/?url=https%3A%2F%2Fpubmed.ncbi.nlm.nih.gov%2F33046362%2F&data=05%7C01%7Crpepe%40rwjms.rutgers.edu%7C24ad09d723384e3acab108daa226a743%7Cb92d2b234d35447093ff69aca6632ffe%7C1%7C0%7C638000584137711691%7CUnknown%7CTWFpbGZsb3d8eyJWIjoiMC4wLjAwMDAiLCJQIjoiV2luMzIiLCJBTiI6Ik1haWwiLCJXVCI6Mn0%3D%7C3000%7C%7C%7C&sdata=vifxen9v3x8edqU4kVqsoVCSf5VQ9aM%2Fn5RWLXx79IM%3D&reserved=0) by Song et al, which showed opioid consumption 72 hours after operation for the ESP block and control groups were 0.945 ± 0.605 mg/kg *v* 1.247 ± 0.184 mg/kg [p = 0.36]). Standard deviations are thus 0.605 and 0.184 for the ESP and control groups, respectively.

For this pilot project the investigators anticipate consenting 150 subjects to obtain 96 evaluable subjects;

48 Mini thoractomies (N=24 Exparel, N=24 Marcaine)

48Open sternotomies (N=24 Exparel, N=24 Marcaine).

Enrolling a 96-patient sample for this initial pilot study is justified in that this sample will be sufficient to generate preliminary data and to prove feasibility under the existing study protocol. At this benchmark, the investigators anticipate that a statistically significant difference will not be detectible; however, by simply expanding enrollment to a larger cohort and including the existing patients in the later analysis, efficiency will be optimized while minimizing waste of valuable research resources.

Pacira Pharmaceuticals, Inc is providing funds for this study but is not a “sponsor”, Pacira’s justification for increasing the number of subjects; “sample required for comparing in mean of opioid consumption 72 hr after treatment between 2 groups is 47 per group after adjusting for 20% drop out rate (total sample size: 94).

This is the information used for sample size calculation:

Outcome measure: opioid consumption 72 hours after treatment

Planned analysis method: 2 samples (2-sided) T test assuming unequal variance.

Alpha=0.05

Beta=0.2 (power =80%)

Effect size: 0.302 (=1.247-0.945)

Sd for tx : 0.605, Sd for control: 0.185

Expected drop out: 20%

# of group: 2 (As a primary objective, only two groups may be considered in sample size calculation. Comparing between bilateral arms unilaterally as a secondary objective is possible by stratification in randomization scheme.)

To obtain equal numbers in each group the investigators will enroll 96 subjects.

**1.6 Study Variables**

**A. Independent Variables, Interventions, or Predictor Variables**

Current standard of care for patients undergoing cardiac surgery at RWJUH is to have field block using Exparel (FDA approved for this indication). For this study subjects will be randomized to receive Exparel or Marcaine (also FDA approved for this indication). The independent variables are the treatments: Exparel in saline versus Marcaine in saline. History of chronic pain or chronic reliance on pain medication will be controlled for, along with other demographic and clinical baseline characteristics, through multivariate regression analysis.

**B. Dependent Variables or Outcome Measures**

**Primary Outcome:**

Postoperative opioid consumption

**Secondary Outcomes:**

**Postoperative nonopioid analgesic consumption**

Postoperative pain scores

Postoperative complications and outcomes (composite of all-cause mortality and major morbidity)

Postoperative qualify of life with SF-12

Hospitalization costs

- 1. **Drugs/Devices/Biologics**

1. **Schedule and Administration**

The study medication (Exparel or Marcaine) will be administered per standard of care via Ultrasound-Guided Erector Spinae Plane (ESP) Blocks, bilaterally for sternotomy and unilaterally for mini-thoracotomy. The drug will be administered prior to cardiac surgery. The dosage will be determined by patient body weight.

1. **Drug/Device Accountability and Storage Method**

Both Exparel and Marcaine are stocked and stored at RWJUH pharmacy located at 1 RWJ Place, New Brunswick, NJ 08901will be prepared, dispensed, disposed of and accounted for per RWJUH’s pharmacy standard practice.

**Specimen Collection** N/A

**1.9 Data Collection**

**Primary Data Collection**

- Research staff (listed on eIRB) will collect information from the subject’s medical record (Epic) and RWJUH Decision Support Service (costs)
- Postoperative pain scores, opioids consumed, and nonopioids consumed will be collected prospectively as a part of routine clinical care and stored in Epic. Opioid doses will be converted to morphine equivalents. Pain scores, morphine equivalents, and the type and quantity of nonopioid medications consumed will be averaged in the first five postoperative days or until discharge (whichever is later) and again at the follow up visit.
- SF-12 questionnaires will be completed by subjects before surgery and at follow up. (Takes < 5 minutes to complete)

Above data will be entered into the Redcap study database (Redcap is database program purchased by Rutgers for use in research. The study database is maintained on the secure RWJMS server, password protected and only accessible to the designated research staff.)

Upon completion of study all identifiers in the database will be removed and stored by study specific ID only. Once this is done there will be no link between subject and health information.

De-identified data will be stored for 6 years in accordance with Rutgers requirements.

**2.0 Project Management**

**2.1 Research Staff and Qualifications**

All investigators listed in eIRB are CITI trained. PI and each coordinator have > 25 years’ experience conducting clinical trials.

- 1. **Research Staff Training**

The PI has met with study staff while developing the study. Prior to starting, the PI will meet with study staff to review the protocol, procedures and each one’s responsibilities going forward. Study progress will be reviewed at monthly meetings.

2.3 Other Resources N/A

**2.4 Research Sites**

Rutgers Robert Wood Johnson Medical School (RWJMS)

Robert Wood Johnson University Hospital (RWJUH)

**3.0 Multi-Center Research**

N/A

**4.0 Subject Considerations**

**4.1 Subject Selection and Enrollment Considerations**

1. **Method to Identify Potential Subjects / B. Recruitment Details**

Potential subjects will be recruited from the Cardiac Surgery Clinic and/or Service (in-patients) day(s) or weeks prior to surgery. The PI or Study Nurse will review patient’s chart for eligibility. Study investigator will discuss the study with the subject. If subject is interested, they will proceed with the consent process.

1. **Subject Screening**

**Inclusion Criteria**

- Adults (18– no upper age limit)
- Scheduled for mini thoracotomy (i.e. valve repair) or open sternotomy (i.e. bypass graft) at RWJUH (in and out-patients).

**Exclusion Criteria**

Patients will be excluded if they;

- - Are currently on pain medication or pain regimen for chronic pain
  - Convert to sternotomy(for thoracotomies)
  - Require, upon intraoperative discovery and surgeon’s decision, the need for an unplanned secondary procedure other than the originally scheduled index operation
  - Emergent surgery

Non-English speaking (The majority of the PI's patient population speak English. As a pilot study the investigators cannot afford to enroll non-English speaking subjects due to time, personnel and financial constraints.

- - Currently on mechanical circulatory support
  - On vasoactive medications,
  - intubated
  - active infection
  - patients otherwise deemed ineligible for ESP block due to safety concerns by the investigators.

**4.2 Obtaining Identifiable Information About** **Non-Subjects**

N/A

**4.3 Number of Subjects**

**A. Total Number of Subjects**

For this pilot project the investigators anticipate consenting 150 subjects to obtain 96 evaluable subjects;

48 Mini thoractomies (N=24 Exparel, N=24 Marcaine)

48Open sternotomies (N=24 Exparel, N=24 Marcaine)

**B. Total Number of Subjects If Multicenter Study**

N/A

**C. Feasibility**

In the past year more the 1500 cardiac surgeries were performed at RWJUH in New Brunswick. The medications involved in this study are readily available and utilized as standard of care.

**4.4** **Consent Procedures**

**A. Consent Process**

**A. Consent Process / Documenting Consent**

- **Individual Roles for Researchers Involved in Consent**

Study MDs or Nurses listed on eIRB will identify potential subjects from the:

- **Location of Consent Process**

1. Cardiac Surgery Clinic. He/she will discuss the study (5-10 mins.) with the subject and give them the written consent to read. After reading the consent, all questions will be answered (as much time as necessary), written consent obtained, and a copy given to the subject. If the subject takes the consent home to read the study doctor or nurse will follow up and if subject wants to participate, written consent will be obtained at another clinical appointment (office visit or pre-admission testing). **Protecting Privacy –** Discussion and consent will take place in private clinic room.

2. In-patient Cardiac Surgery Service. Patients will be approached one or more days prior to surgery in the hospital room. MD/RN will discuss the study with the subject (5-10 mins.) and give them the written consent to read. MD/RN will return after allowing ample time for the patient to read the consent. Once read all questions will be answered (as much time as necessary) , written consent obtained, and a copy given to the subject. **Protecting Privacy –** Discussion and consent will take place at the patient’s bedside.

Consent and process will be documented in subject’s medical record as a note by the investigator obtaining consent.

**Ongoing Consent N/A**

**Coercion or Undue Influence** Consent discussion starts with explaining that this study is completely voluntary, and they can say no or stop the discussion at any time.

**Subject Understanding** After all questions have been answered and before signing the consent, the investigator will ask several questions about the study to ensure the subject understands**.**

**Waiver or Alteration of Consent Process** N/A

**4.5** **Special Consent/Populations**

1. **Minors-Subjects Who Are Not Yet Adults** N/A
2. **Wards of the State** N/A
3. **Non-English-Speaking Subjects** N/A
4. **Enrolling Adults Lacking Decision-Making Capacity (Surrogate Consent)** N/A

**4.6 Economic Burden and/or Compensation for Subjects**

**A. Expenses**

Subjects will not incur any costs due to participation in this study. Cost of the medication is included in the surgery cost.

1. **Compensation/Incentives** N/A

**C Compensation Documentation** N/A

**4.7 Risks of Harm/Potential for Benefits to Subjects**

1. **Description of Risks of Harm to Subjects**

**Reasonably Foreseeable Risks of Harm and Minimizing these Risks**

Both medications in this study are FDA approved for this indication. The risks of each drug are very similar. Subjects undergoing cardiac surgery at RWJUH would normally receive Exparel. Therefore the only risk specific to this study is if one medication is less effective at controlling pain then the other. If this occurs, the subject’s pain and/or other side effects will be managed per routine clinical care.

**Common side effects of Marcaine include:**

- nausea,
- vomiting,
- chills or shivering,
- headache,
- back pain,
- dizziness,
- problems with sexual function,
- restlessness,
- anxiety,
- dizziness,
- ringing in the ears,
- blurred vision, or
- tremors.

**Side effects of Exparel include:**

- dizziness,
- drowsiness,
- nausea,
- constipation,
- vomiting,
- itching,
- headache,
- back pain, or
- swelling in hands or feet.

Loss of confidentiality - Every effort will be made to maintain patient confidentiality. Only study personnel will have access to the study data.

1. **Procedures which Risk Harm to Embryo, Fetus, and/or Pregnant Subjects** N/A
2. **Risks of Harm to Non-Subjects** N/A
3. **Assessment of Social Behavior Considerations** N/A
4. **Minimizing Risks of Harm** The only risk specific to this study is if one medication is less effective at controlling pain then the other. If this occurs, the subject’s pain and/or other side effects will be managed per routine clinical care**.**

**F. Potential Direct Benefits to Subjects**

There may be no direct benefit to subjects. Future cardiac patients may benefit from the knowledge gained from this study*.*

**5.0 Special Considerations**

**5.1 Health Insurance Portability and Accountability Act (HIPAA)**

HIPPA language included in consent.

**5.2 Family Educational Rights and Privacy Act (FERPA)** N/A

**5.3 Code of Federal Regulations Title 45 Part 46 (Vulnerable Populations)** N/A

**5.4 General Data Protection Regulation (GDPR)** N/A

**5.5 NJ Access to Medical Research Act (Surrogate Consent)** N/A

**6.0 Data Management Plan**

**6.1 Data Analysis**

The investigators will conduct a Mann-Whitney or t-test (the latter if the data collected is a normal distribution) for continuous variables, and a Fisher’s exact or chi-squared (the former if N<10) for categorical variables. The investigators will initially compare sample descriptive data to scale the range of diversity within and between the two groups.

After describing the sample, the investigators will compare continuous dependent variables (length of stay in the hospital and the ICU, costs, pain scores, and postoperative opioid and non-opioid pain medication use) with Mann-Whitney or t-tests. The investigators will repeat these calculations by operation type.

Opioid use will be quantified by morphine equivalents, as variables will present in mode of administration and type of opioid. The measurements will be taken as a total volume used during the hospital stay and a total volume used during the week of the follow up appointment.

Pain scores will be averaged daily through post-op day 5 and again at the first follow-up appointment (~4 weeks after discharge).

Paired t-test will be performed on SF-12 scores.

**6.2 Data Security**

- Research staff will enter information from the subject’s medical record and SF-12 results into the Redcap study database
- Upon completion of study all identifiers in the database will be removed and stored by study specific ID only. Once this is done there will be no link between subject and health information.
- De-identified data will be stored for 6 years in accordance with Rutgers requirements.

**6.3 Data and Safety Monitoring** N/A

**6.4 Reporting Results**

1. **Subject Results Reporting**

Results will not be shared with individual subjects.

1. **Professional Reporting**

Results will be submitted for presentation and publication in the appropriate surgical or rehabilitation journals, conferences, and forums

**6.4** **Secondary Use of the Data** N/A

1. **Clinical Trials Registration, Results Reporting and Consent Posting**

Once approved, this study will be registered on clinicaltrials.gov.

**6.5 Secondary Use of the Data**

The PI may use this data in the future but no plans to share with other investigators.

**7.0 Research Repositories – Specimens and/or Data**

N/A

**8.0 Approvals/Authorizations**

RUG/IRC form uploaded

**9.0 Bibliography**

1. Toscano A, Barbero C, Capuano P, et al. Chronic postsurgical pain and quality of life after right minithoracotomy mitral valve operations. *J Card Surg*. 2022;37: 1585-90.
2. Nagaraja PS, Ragavendran S, Singh NG, Asai O, Bhavya G, Manjunath N. Comparison of continuous thoracic epidural analgesia with bilateral erector spinae plane block for perioperative pain management in cardiac surgery. *Ann Card Anaesth*. 2018; 21(3):323-7.
3. Macaire P, Ho N, Nguyen T, Nguyen B, Vu V, Quach C, Roques V, Capdevila X. Ultrasound-Guided continuous thoracic erector spinae plane block within an enhanced recovery program is associated with decreased opioid consumption and improved postoperative rehabilitation after open cardiac surgery – a patient-matched, controlled before-and-after study. *J Cardiothorac Vasc Anesth.* 2019;33 (6):1659-67.
4. Song K, Xu Q, Knott VH, Zhao CB, Clifford SP, Kong M, Slaughter MS, Huang Y, Huang J. Liposomal bupivacaine–based erector spinae block for cardiac surgery.*J Cardiothoracic and Vascular Anesthesia.* 2021;35 (5):1555-9.
5. Collins JB, Song J, Mahabir RC. Onset and duration of intradermal mixtures of bupivacaine and lidocaine with epinephrine. Can J Plast Surg. 2013;21(1):51–3.
6. Gadsden J, Long WJ. Time to Analgesia Onset and Pharmacokinetics After Separate and Combined Administration of Liposome Bupivacaine and Bupivacaine HCl: Considerations for Clinicians. Open Orthop J. 2016 Apr 12;10:94–104.
7. Southern Illinois University. A Prospective, Randomized, Single-Blind, Active Control Trial to Compare Safety and Effectiveness Of Liposomal Bupivacaine (Exparel) to Standard Bupivacaine HCl (Marcaine) for Pain Management in Patients Undergoing Video-Assisted Thoracoscopic Lobectomy [Internet]. clinicaltrials.gov; 2021 Nov [cited 2022 Sep 28]. Report No.: NCT03682224. Available from: <https://clinicaltrials.gov/ct2/show/NCT03682224>
8. M.D JDH. Randomized Non-inferiority Trial of Continuous Thoracic Epidural Analgesia Versus Single Intercostal Nerve Block After Thoracotomy [Internet]. clinicaltrials.gov; 2019 Dec [cited 2022 Sep 28]. Report No.: NCT02178553. Available from: <https://clinicaltrials.gov/ct2/show/NCT02178553>
9. Sandhu HK, Miller CC III, Tanaka A, Estrera AL, Charlton-Ouw KM. Effectiveness of Standard Local Anesthetic Bupivacaine and Liposomal Bupivacaine for Postoperative Pain Control in Patients Undergoing Truncal Incisions: A Randomized Clinical Trial. JAMA Network Open. 2021 Mar 16;4(3):e210753.
10. Song K, Xu Q, Knott VH, Zhao CB, Clifford SP, Kong M, et al. Liposomal Bupivacaine-Based Erector Spinae Block for Cardiac Surgery. J Cardiothorac Vasc Anesth. 2021 May;35(5):1555–9.
